# Fibrolysin in Meniere’s Disease

**Published:** 1914-11

**Authors:** 


**Fibrolysin in Meniere's Disease.** Soca of Montivideo, Bull. de la Soc. Med. des Hop., 1913, No. 35, reports 18 successful cases. One ampoule, 2.3 c.c. was administered daily for ten days, the treatment being repeated after an intermission of 10 days, or an injection every other day according to the severity and results. The promptness of results in some cases and success in a case of leukemic vertigo without evidence of sclerosis, suggest that the action is due to some systemic effect and not to histolysis.

